# The Effectiveness of the Modified Side-Locking Loop Suture Technique with Early Accelerated Rehabilitation for Acute Achilles Tendon Rupture in Athletes

**DOI:** 10.3390/jcm13195818

**Published:** 2024-09-29

**Authors:** Yuta Matsumae, Shota Morimoto, Masashi Nakamura, Futoshi Morio, Tomoya Iseki, Toshiya Tachibana

**Affiliations:** 1 Department of Orthopaedic Surgery, Hyogo Medical University, 1-1, Mukogawa-cho, Nishinomiya 663-8501, Hyogo, Japan; 2 Department of Orthopaedic Surgery, Osaka Minato Central Hospital, 1-7-1, Isoji-cho, Osaka 552-0003, Osaka, Japan; 3 Department of Orthopaedic Surgery, Takarazuka City Hospital, 4-5-1, Kohama, Takarazuka 665-0827, Hyogo, Japan

**Keywords:** Achilles tendon rupture, operative treatment, early accelerated rehabilitation, modified side-locking loop suture technique, athletes

## Abstract

**Background/Objectives:** An early accelerated rehabilitation is generally recommended after surgery for acute Achilles tendon ruptures (ATRs). The modified side-locking loop suture (MSLLS) is a surgical technique that provides high tensile strength to the repaired Achilles tendon and allows for a safe, early accelerated rehabilitation protocol without requiring postoperative immobilization. However, there are no reports investigating the clinical outcomes of the MSLLS technique with early accelerated rehabilitation for ATRs. To clarify the effectiveness of the MSLLS technique with an early accelerated rehabilitation protocol for ATR in athletes. **Methods:** We retrospectively analyzed 27 athletes (Tegner activity score ≥ 6) who underwent surgical treatment using the MSLLS technique for ATR between April 2017 and August 2022. All patients underwent an early accelerated rehabilitation protocol without immobilization. Outcome measures included the American Orthopaedic Foot and Ankle Society Ankle-Hindfoot Scale (AOFAS) score taken preoperatively and one year postoperatively, the time required to perform 20 continuous double-leg heel raises (DHR) and single-leg heel raises (SHR), the time to return to the original sport, and the presence of any complications. **Results:** The mean AOFAS score significantly improved from 37.2 ± 9.7 preoperatively to 96.3 ± 5.3 one year postoperatively. The mean time to be able to perform 20 continuous DHR and SHR was 7.7 ± 1.2 weeks and 11.3 ± 1.6 weeks, respectively. All patients were able to return to their original sport at their pre-injury level in an average of 22.7 ± 3.7 weeks without complication. **Conclusions:** The MSLLS technique in conjunction with an early accelerated rehabilitation protocol for ATR in athletes produced satisfactory results, with all patients able to return to their original sport at their preinjury level without complication.

## 1. Introduction

Achilles tendon ruptures (ATRs) occur most often in male athletes who are in their 30s and 40s [1,2]. Recent systematic reviews have shown a 76% return-to-play (RTP) rate with an average RTP duration of 11 months in high-level athletes, while non-elite athletes had an 80% RTP rate and an average RTP duration of 6 months [3,4]. These results are not satisfactory for athletes at any level, and ATRs can be devastating to athletes. Therefore, various studies have been conducted on the outcomes of ATR.

Treatment of ATRs is generally divided into conservative and surgical treatment, but the optimal treatment has not yet been determined [5,6]. Conventionally, late rehabilitation protocols of several weeks of immobilization and non-weight-bearing have been applied for both forms of treatment [7,8]. However, several recent studies have shown that an early accelerated rehabilitation protocol that includes early weight-bearing and early range of motion exercises can have a beneficial effect on the healing process of the ruptured Achilles tendon [5,7,9,10,11,12,13]. These studies suggest that early accelerated rehabilitation protocols may result in quicker recovery of the tendon tissue’s functional properties, enabling earlier return to work and sports compared to conventional rehabilitation protocols [5]. Therefore, an early accelerated rehabilitation protocol is generally recommended for either conservative or surgical treatment of ATRs.

Recently, various operative techniques for ATRs have been reported. The side-locking loop suture technique is one of these operative techniques and was first introduced by Yotsumoto et al. in 2010 [13]. This technique, which consists of four side-locking points and one suturing site placed on the distal stump of the tendon, provides higher tensile strength to the suture site than other surgical procedures and allows for a safe, early rehabilitation protocol without requiring postoperative immobilization. However, in the SLLS technique, it can be difficult for surgeons to adjust the tensile force at the Achilles tendon suture site because each side-locking point does not easily slide. To resolve this issue, Imade et al. devised the modified side-locking loop suture (MSLLS) technique, which is an improvement of the original SLLS technique [14]. However, to our best knowledge, there have been no reports investigating the clinical outcomes of the MSLLS technique in combination with an early accelerated rehabilitation protocol for ATRs.

The purpose of this study was to review our experiences using the MSLLS technique in conjunction with an early accelerated rehabilitation protocol for ATR in athletes. It was hypothesized that this approach would yield superior outcomes compared to other treatment options in previous relevant studies.

## 2. Materials and Methods

This study included 30 athletes with a Tegner activity score ≥ 6 who underwent surgical treatment using the MSLLS technique for ATR between April 2017 and August 2022. Postoperatively, an early accelerated rehabilitation protocol without immobilization was applied in all patients. The senior author (S.M.) performed all surgeries and provided pre- and postoperative management for all patients. The diagnosis of ATR was based on physical findings, such as palpation of the defect at the rupture site, loss of tendon relief, and positive Simmonds–Thompson test. In addition, plain lateral radiographs of the affected ankle were taken in all cases to rule out avulsion fractures of the calcaneus. Patients with a postoperative follow-up period of less than one year and patients with a history of surgery on the affected lower extremity were excluded. This study was approved by our institutional ethics review board (No. 4655) and written informed consent was obtained from all patients.

### 2.1. Surgical Techniques

**Figure 1:**
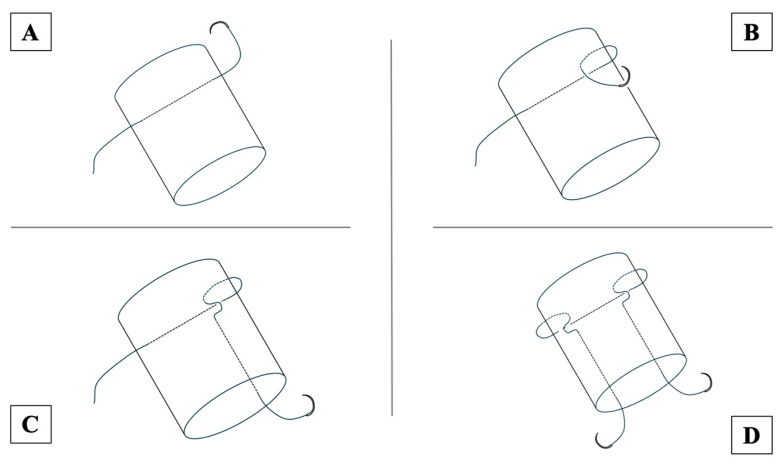
The illustration shows how to make two side-locking loop points for the proximal stump of the Achilles tendon using USP No. 5 braided polyblend suture thread. (**A**) First, the suture thread is passed perpendicularly to the tendon, approximately 3 cm from the proximal stump. (**B**) The suture is passed from behind the tendon to the front slightly proximal to the transverse suture (**C**) and then passed from the front of the tendon slightly distal to the transverse suture to the proximal end through the tendon, making the side-locking point. (**D**) Two side-locking points are created on the medial and lateral sides of the tendon.

**Figure 2:**
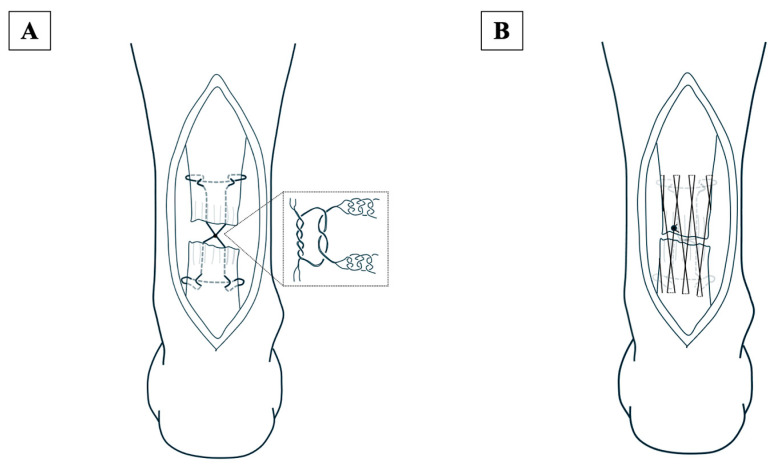
(**A**) The sutures from the proximal and distal stumps are tied between the stumps using an antislip knot as a core suture, keeping the operated ankle in a neutral position. (**B**) After the core suture, a peripheral suture is made using the cross-stitch method with a USP No. 2-0 monofilament nylon suture.

All surgeries were performed in the prone position under general or lumbar anesthesia. An air tourniquet was used in all cases. A skin incision of approximately 7 cm was made slightly medial to the midline of the Achilles tendon, followed by a fascial incision of the same length. The paratenon was then carefully dissected from the tendon. After the rupture was confirmed, the proximal stump of the Achilles tendon was sutured using USP No. 5 braided polyblend suture thread (Fiber Wire, Arthrex Inc., Naples, FL, USA), creating two side-locking loop points, one medial and one lateral, at approximately 3 cm from the stump (Figure 1). The distal stump of the Achilles tendon was sutured in the same manner. The proximal and distal stump sutures were tied between the stumps using an antislip knot technique as a core suture, with the operated ankle in a neutral position (Figure 2A). After the core suture, a peripheral suture was made using the cross-stitch method with a USP No. 2-0 monofilament nylon suture (Figure 2B). Finally, the paratenon and fascia were repaired over the sutured Achilles tendon, and the subcutaneous tissue and skin were closed.

### 2.2. Postoperative Rehabilitation Protocol and Follow-Ups

Postoperative immobilization was not applied, and active and passive range of motion exercises of the operated ankle were immediately initiated the day after surgery. When the active dorsiflexion of the operated ankle was 0° or more, partial weight-bearing was allowed. Full weight-bearing without crutches began at 4 weeks postoperatively. Double-leg heel raises (DHR) and muscle-strengthening exercises were initiated at 6 weeks postoperatively, and single-leg heel raises (SHR) exercises were started when the patients were able to perform DHR without support. Jogging was permitted at 8 weeks postoperatively, and competition-specific exercises were allowed once the patients were able to perform 20 continuous SHR.

The patients were followed up every 2 weeks until they returned to their original sport. Thereafter, they were followed up at 3-month intervals. 

### 2.3. Clinical Evaluation

Clinical subjective evaluation was performed using the American Orthopaedic Foot and Ankle Society Ankle-Hindfoot Scale (AOFAS) score, taken preoperatively and one year postoperatively. The time to be able to perform 20 continuous DHR and 20 continuous SHR, the time to return to the original sport, and the presence of any complications were also recorded. Time to return to the original sport was defined as the time between surgery and return to the original sport at the preinjury level of performance.

### 2.4. Statistical Analysis

Statistical analysis was performed using SPSS software (Version 19; IBM: Armonk, NY, USA). A *p* value < 0.05 was considered statistically significant. Comparison of pre- and postoperative AOFAS scores was performed using a paired *t* test.

## 3. Results

Of the 30 patients initially enrolled, two patients with less than one year of follow-up and one patient with a history of previous surgery on the affected lower extremity were excluded from the study, and the remaining 27 patients were analyzed. The 27 patients consisted of 21 male and 6 female athletes, with a mean age of 35.8 ± 10.3 years (range, 20–54 years) at the time of surgery and a mean Tegner activity score of 6.7 ± 1.1 (range, 6–9). Thirteen athletes were injured during soccer, four during basketball, three during volleyball, two during judo, two during kendo, and three during other sports. The average follow-up period was 32.4 ± 9.1 months (range, 12–60 months). Patient demographics from this study are presented in Table 1.

The mean AOFAS score improved significantly from 37.2 ± 9.7 preoperatively to 96.3 ± 5.3 one year postoperatively (*p* < 0.001). The average time it took each patient to perform 20 continuous DHR and 20 continuous SHR was 7.7 ± 1.2 weeks and 11.3 ± 1.6 weeks, respectively. All patients were able to return to their original sport at their pre-injury level in an average of 22.7 ± 3.7 weeks. There were no reported complications. The clinical outcomes of this study are presented in Table 2.

## 4. Discussion

The present study shows that surgical treatment using the MSLLS technique with an early accelerated rehabilitation protocol could provide satisfactory results in athletes with ATRs, enabling all the athletes to return to their original sport at their preinjury level without complication.

Treatment of ATRs can be divided into conservative and surgical treatment; however, the optimal method has yet to be determined [5,6]. Conventionally, late rehabilitation protocols with several weeks of non-weight-bearing and immobilization have been applied for both conservative and surgical treatment [7,8]. In recent years, several animal and clinical studies have suggested that an early rehabilitation protocol can have a beneficial effect on the healing process of the Achilles tendon [5,10,15,16]. These studies indicate that an early rehabilitation protocol may restore the functional properties of tendon tissue more quickly than a traditional rehabilitation protocol, leading to an earlier return to work and sports [5]. Several systematic reviews have also shown that an early rehabilitation protocol has advantages, including lower rerupture rates, increased patient satisfaction, and improved function [17,18]. Therefore, an early rehabilitation protocol is generally recommended regardless of the type of treatment. In addition, studies comparing clinical outcomes of conservative and surgical treatment with early rehabilitation protocols suggest that the incidence of rerupture and patient-reported outcome scores are similar for both treatment modalities [5,10,19]. On the other hand, Soroceanu et al. found that a return to sports and work was faster with surgical treatment compared to conservative treatment, although rerupture rates, muscle strength, and calf circumference were comparable between the two treatments [5]. Furthermore, Lantto et al. have demonstrated that conservative treatment results in less muscle strength at 18 months postoperatively compared to surgical treatment [20]. For this reason, several orthopaedic surgeons recommend surgical treatment with an early rehabilitation protocol as the preferred treatment for athletes with ATRs.

The SLLS method is one of several surgical techniques and was first introduced by Yotsumoto et al. in 2010 [13]. This technique, which consists of four side-locking points and one suturing site placed on the distal stump of the tendon, provides higher tensile strength to the suture site than other surgical procedures and allows for a safe, early rehabilitation protocol without requiring postoperative immobilization. Yotsumoto et al. reported favorable clinical outcomes in 20 ATR patients treated with the SLLS technique and an early rehabilitation protocol, with an average time of 6.3 weeks and 9.9 weeks before they were able to complete 20 continuous DHR and 20 continuous SHR, respectively. The average time to return to work or sports was 14.4 weeks. One patient, however, had difficulty dorsiflexing the operated ankle. In the original SLLS technique, it is difficult for surgeons to adjust the tensile force at the Achilles tendon suture site because each side-locking point does not easily slide [13]. To resolve this issue, two operative techniques such as the double SLLS and the MSLLS technique have been reported [14,21]. Miyamoto et al. introduced the double SLLS technique that improved the original SLLS technique [21]. In this technique, the proximal end of the tendon is firstly sutured with a 2-strand SLLS technique using USP 2 size braided polyblend suture thread. In the same manner, the proximal end is sutured using another USP 2 size braided polyblend suture thread, resulting in a 4-strand SLLS technique with two suture threads. Next, the distal end is sutured with a 4-strand SLLS technique using two suture threads in the same manner for the proximal end. Thereafter, these sutures are tied between the proximal and distal ends using the antislip knot technique. Miyamoto et al. analyzed 44 athletes with ATR who were treated with the double SLLS technique and an early rehabilitation protocol. They have demonstrated that all athletes, except one who was a classic ballet dancer, could return to athletic activities with an average time of 17.1 weeks. In addition, Imade et al. devised the MSLLS technique, which is an improvement of the original SLLS technique [14]. In the MSLLS technique, the proximal and distal ends of the tendon are each sutured with a 2-strand SLLS technique using USP 5 size braided polyblend suture thread, and these sutures are tied between the proximal and distal ends using the antislip knot technique. The ultimate tensile strength of the MSLLS method is about 630–650 N, which is considerably less than the original SLLS technique of approximately 900 to 950 N [13]. Considering the tensile strength of the Achilles tendon ranges from 489 to 661 N while riding a bicycle [22], the tensile strength of the MSLLS technique is sufficient to safely enable an early accelerated rehabilitation protocol without requiring postoperative immobilization. However, there have been no reports investigating the clinical outcomes of the MSLLS technique in combination with an early accelerated rehabilitation protocol for athletes with ATRs.

In this study, surgical treatment using the MSLLS technique and an early rehabilitation protocol yielded satisfactory outcomes and all patients were able to return to sports at an average of 22.7 weeks without complications. Considering that the activity level of the patients in this study was higher than those reported by Yotsumoto et al. [13], the results of the present study were at least comparable or superior to those of the original SLLS technique for ATRs. In addition, the MSLLS technique has the advantage that the tensile force at the tendon suture site can be easily adjusted similar to the double SLLS technique [14,21]; there were no patients who had difficulty dorsiflexing the operated ankle in the present study. Furthermore, compared to the double SLLS technique, the MSLLS technique has some advantages such as an easier process of suturing the tendon and lower medical costs due to the smaller amount of braided polyblend suture thread used for surgery. However, the mean time to return to the original sport in the study by Miyamoto et al. was 17.1 weeks [21], which was slightly earlier than the present study results with 22.7 weeks. In the report by Miyamoto et al., return to the original sport was defined as “return to athletic activities”, whereas it was defined as “return to the original sport at the preinjury level of performance” in our report. Considering this difference, statistical comparison is not feasible, and the present study results seem at least comparable with those of the study by Miyamoto et al.

This study has several limitations. First, this is a retrospective case series. Second, the sample size is small. Third, this study had no control group comparing the MSLLS technique with other operative techniques. Furthermore, while a longer follow-up period is generally needed to clarify the treatment outcomes of ATRs, the patients in this study were followed for only a short period of time.

## 5. Conclusions

The MSLLS technique in conjunction with an early accelerated rehabilitation protocol for athletes with ATRs produced satisfactory results, allowing all patients to return to their original sport at their preinjury level without complications. Although further studies with a larger sample size, a longer follow-up, and more rigorous designs may be required, this treatment procedure may be a useful option for athletes with ATR.

## Figures and Tables

**Table 1:** Patient demographics of the study.

Age, years *	35.8 ± 10.3
Sex, male/female, n	21/6
Side, right/left, n	10/17
Sport, n	
soccer	13
basketball	4
volleyball	3
judo	2
kendo	2
others	3
Tegner activity score *	6.7 ± 1.1
Follow-up period, months *	32.4 ± 9.1

* Values are expressed as mean ± SD.

**Table 2:** Clinical outcomes.

AOFAS score, points	
Pre-operation *	37.2 ± 9.7
One year after operation *	96.3 ± 5.3
*p* value	<0.001
Time to be able to perform 20 continuous DHR, weeks *	7.7 ± 1.2
Time to be able to perform 20 continuous SHR, weeks *	11.3 ± 1.6
Time to return to original sport, weeks *	22.7 ± 3.7

* Values are expressed as mean ± SD.

## Data Availability

The data presented in this study are available in the article.
